# Expression of wild-type *PtrIAA14.1*, a poplar Aux/IAA gene causes morphological changes in *Arabidopsis*

**DOI:** 10.3389/fpls.2015.00388

**Published:** 2015-06-02

**Authors:** Shanda Liu, Qingnan Hu, Sha Luo, Qianqian Li, Xiyu Yang, Xianling Wang, Shucai Wang

**Affiliations:** Key Laboratory of Molecular Epigenetics of Ministry of Education, Northeast Normal University, Changchun, China

**Keywords:** Aux/IAA, auxin response factor, PtrIAA14.1, vascular patterning, *Arabidopsis*, *Populus trichocarpa*

## Abstract

Aux/IAA proteins are transcriptional repressors that control auxin signaling by interacting with auxin response factors (ARFs). So far all of the identified Aux/IAA mutants with auxin-related phenotypes in *Arabidopsis* and rice (*Oryza sativa*) are dominant gain-of-function mutants, with mutations in Domain II that affected stability of the corresponding Aux/IAA proteins. On the other hand, morphological changes were observed in knock-down mutants of *Aux/IAA* genes in tomato (*Solanum lycopersicum*), suggesting that functions of Aux/IAA proteins may be specific for certain plant species. We report here the characterization of PtrIAA14.1, a poplar (*Populus trichocarpa*) homolog of IAA7. Bioinformatics analysis showed that PtrIAA14.1 is a classic Aux/IAA protein. It contains four conserved domains with the repressor motif in Domain I, the degron in Domain II, and the conserved amino acid signatures for protein–protein interactions in Domain III and Domain IV. Protoplast transfection assays showed that PtrIAA14.1 is localized in nucleus. It is unable in the presence of auxin, and it represses auxin response reporter gene expression. Expression of wild-type *PtrIAA14.1* in *Arabidopsis* resulted in auxin-related phenotypes including down-curling leaves, semi-draft with increased number of branches, and greatly reduced fertility, but expression of the *Arabidopsis Aux/IAA* genes tested remain largely unchanged in the transgenic plants. Protein–protein interaction assays in yeast and protoplasts showed that PtrIAA14.1 interacted with ARF5, but not other ARFs. Consistent with this observation, vascular patterning was altered in the transgenic plants, and the expression of *AtHB8* (*Arabidopsis thaliana homeobox gene 8*) was reduced in transgenic plants.

## Introduction

Auxin regulates most, if not all, aspects of plant growth and development, such as organ formation, lateral root initiation, stem and root elongation, vascular tissue differentiation, and apical dominance (Davies, 1995). Auxin-regulated plant growth and development is likely initiated by the rapid response of specific genes to local changes in auxin concentration. These genes are referred to as early auxin response genes. *Aux/IAA* genes are one of the several different types of early auxin response genes in *Arabidopsis* (Guilfoyle, 1999; Hagen and Guilfoyle, 2002).

There are 29 Aux/IAA genes in the *Arabidopsis* genome (Reed, 2001; Liscum and Reed, 2002). Aux/IAA proteins are transcription repressors that involve in the regulation of auxin signaling. Based on their amino acid sequence similarity, Aux/IAA proteins generally contain four conserved domains, Domain I, II, III, and IV. Domain I contains a conserved LxLxL motif that functions as an active repression domain. Domain II contains a conserved degron that regulates the stability of Aux/IAA proteins in an auxin-dependent manner. Domains III and IV are related to the conserved C-terminal dimerization domains of auxin response factors (ARFs), and contain conserved amino acids that are required for homo and hetero interactions among Aux/IAA proteins and ARFs (Ulmasov et al., 1997, 1999; Ramos et al., 2001; Tiwari et al., 2001, 2003, 2004; Dreher et al., 2006; Nanao et al., 2014).

Based on the results obtained largely from *Arabidopsis* protoplast transient transfection and yeast two-hybrid assays, it was proposed that when auxin concentrations are low, Aux/IAA repressors are dimerized with ARF activators that bound on the TGTCTC auxin response elements (AuxREs) in the promoter regions of auxin response genes, resulting in repression of auxin response genes. When auxin concentrations are elevated, Aux/IAA repressors are degraded by the ubiquitin–proteasome pathway, allowing the ARF activators to activate auxin response genes (Ulmasov et al., 1999; Tiwari et al., 2001, 2004; Guilfoyle and Hagen, 2007).

Functions of *Aux/IAA* genes in regulating plant growth and development are largely revealed by characterization of dominant gain-of-function mutations in Domain II of Aux/IAA proteins. Several different types of phenotypes were observed in the dominant *iaa* mutants (Nagpal et al., 2000; Rogg et al., 2001; Fukaki et al., 2002; Uehara et al., 2008; Ploense et al., 2009; Rinaldi et al., 2012), and some of the dominant gain-of-function *iaa* mutants even have opposite phenotypes (Tian and Reed, 1999; Tian et al., 2002; Knox et al., 2003). Promoter-exchange experiments further confirmed that Aux/IAA genes have both specific and similar functions (Muto et al., 2007). On the other hand, all single T-DNA insertion mutants identified in *Arabidopsis* so far, and double or triple mutants of closely related Aux/IAA genes in *Arabidopsis* showed no visible developmental defects (Overvoorde et al., 2005).

Consistent with the phenotypes observed in the T-DNA insertion and dominant gain-of-function Aux/IAA mutants, *Arabidopsis* transgenic plants expressing glucocorticoid hormone-binding domain (GR) fused protein of wild-type IAA1 (IAA1-GR) did not exhibit any obvious phenotypic differences upon DEX treatment (Park et al., 2002). Transgenic *Arabidopsis* plants expressing wild-type *VvIAA19*, a grape (*Vitis vinifera*) Aux/IAA gene, are also morphological similar to wild-type plants (Fujita et al., 2012; Kohno et al., 2012). However, auxin-related phenotypes were observed in DEX treated transgenic plants expressing GR fused IAA1 with a mutation in Domain II (iaa1-GR; Park et al., 2002), and transgenic plants expressing *mIAA14-GR* under the control of the stele-specific *SHORT-ROOT* promoter (Fukaki et al., 2005). Transgenic *Arabidopsis* plants expressing IAA20 and IAA30, two Aux/IAA proteins lacking Domain II, and IAA31, an Aux/IAA with a partial, dominant mutation-type domain II, also showed auxin-related phenotypes (Sato and Yamamoto, 2008).

Similar to the observations in *Arabidopsis*, the Aux/IAA mutants with auxin-related phenotypes isolated in rice (*Oryza sativa*), including *osiaa11*, *osiaa13*, and *osiaa23* were all caused by dominant gain-of-function mutations in the Domain II (Jun et al., 2011; Kitomi et al., 2012; Zhu et al., 2012), and auxin-related phenotypes were observed in transgenic rice plants expressing *OsIAA4*, an Aux/IAA with a dominant mutation-type domain II (Song and Xu, 2013).

In tomato (*Solanum lycopersicum*), however, phenotypic changes were observed in knock-down mutants of Aux/IAA genes (Wang et al., 2005a; Bassa et al., 2012; Deng et al., 2012; Su et al., 2014), suggesting that functions of Aux/IAA proteins may be specific for particular plant species.

There are a total of 35 genes in poplar (*Populus trichocarpa*) encoding Aux/IAA proteins (Kalluri et al., 2007), but their functions remain largely unknown. Because IAA7 is a classic Auxin/IAA protein and its roles in auxin signaling and auxin-regulated plant growth and development have been well characterized in *Arabidopsis* (Nagpal et al., 2000; Muto et al., 2007), we decided to use IAA7 homolog in poplar as an example to study the roles of poplar Aux/IAA proteins in auxin signaling and plant growth and development.

Using the full length amino acid sequence of IAA to BLAST search poplar protein database (www.phytozome.net), we identified PtrIAA14.1 as a poplar homolog of *Arabidopsis* IAA7. We showed that PtrIAA14.1 regulates auxin response reporter gene expression in protoplasts, and that expressing wild-type PtrIAA14.1 in *Arabidopsis* resulted in auxin-related phenotypes.

## Materials and Methods

### Plant Materials and Growth Conditions

*Arabidopsis thaliana* (*Arabidopsis*) ecotype Columbia (Col-0) was used for protoplasts isolation and plant transformation. *DR5:GUS* transgenic plants were used for crossing and protoplasts isolation, and leaves from field-grown poplar (*P. trichocarpa*) tree were used for *PtrIAA14.1* gene cloning and expression analysis.

For auxin treatment and RNA isolation from seedlings, seeds were sterilized and sown on plates containing 0.6% (w/v) phytoagar solidified 1/2 Murashige & Skoog (MS) medium with vitamins (PlantMedia) and 1% (w/v) sucrose. After kept at 4°C in darkness for 2 days, the plates were moved into a growth room. For plant transformation and phenotypic analysis for adult plants, *Arabidopsis* seeds were sown directly into soil and kept in a growth room. *Arabidopsis* plants were grown at 20°C, with a 16 h light/8 h darkness photoperiod at approximately 120 μmol m^–2^ s^–1^.

### Constructs

The *LexA-Gal4-GUS* reporter, and *GD*, *LD-VP*, and *CAT* constructs used for protoplast transfection were described previously (Tiwari et al., 2003; Wang et al., 2007).

To generate HA or GD tagged constructs for *PtrIAA14.1* for protoplast transfection assays, the full-length open-reading frame (ORF) of *PtrIAA14.1* was amplified by RT-PCR using RNA isolated from the poplar leaves, and the PCR products were cloned in frame with an N-terminal HA or GD tag into the *pUC19* vector under the control of the double *35S* enhancer promoter of *CaMV* (Tiwari et al., 2003; Wang et al., 2005b). For plant transformation, the *pUC19HA-PtrIAA14.1* construct was digested with proper enzymes, and subcloned into the binary vector *pPZP211* (Hajdukiewicz et al., 1994) to generate binary vector *pPZP211HA-PtrIAA14.1*.

To generate GD tagged PtrIAA14.1CTD for protoplast transfection, ORF sequences corresponding to the amino acid residues 131–248 of PtrIAA14.1 were amplified by PCR using *pUC19HA-PtrIAA14.1* plasmids as template, and cloned in frame with an N-terminal GD tag into the *pUC19* vector.

To generate GFP tagged PtrIAA14.1 for protoplast transfection, ORF of PtrIAA14.1 without stop codon were amplified by PCR using *pUC19HA-PtrIAA14.1* plasmids as template, and cloned in frame with an C-terminal GFP tag into the *pUC19* vector.

To generate *pDHB1ARF5CTD* construct for yeast-two-hybrid assays, ORF sequences corresponding to the amino acid residues 767–901 of ARF5 were amplified by PCR using *pUC19HA-ARF5* (Wang et al., 2005b) plasmids as template, and cloned into *pDHB1* vector. *pPR3-NPtrIAA14.1* were generated by cloning PCR amplified PtrIAA14.1 full length ORF into *pPR3-N* vector.

All primers used for gene cloning and gene expression analysis are listed in Table 1.

**TABLE 1:** **List of primers used in this study**.

**Gene**	**Primer**	**Sequence (5′–3′)**
*PtrIAA14.1*	Forward Reverse	CAACATATGACAACTTCTGTGCTAGG CAAGAGCTCTCAGGTTCTGCTTTCGC
*PtrIAA14.1 CTD*	Forward Reverse	CAACATATGGCATTTGTGAAGGTC CAAGAGCTCTCAGGTTCTGCTTTCGC
*PtrIAA14.1 SfiI*	Forward Reverse	GGCCATTACGGCCATGACAACTTCTGTGCTAGG GGCCGAGGCGGCCAGGTTCTGCTCTTGCATTTC
*UBQ*	Forward Reverse	CTCAAAGTGAAAGGCCAGGATG ACTGTCAAAGCTCTTGGTGAG
*IAA1*	Forward Reverse	AACGACTCAACAGAAGAATCTGC CACATAACTCACGTTTTTGTTGTTG
*IAA2*	Forward Reverse	CTATTTGAGGAAACTCGTGATGAAG TCACGTAGCTCACACTGTTG
*IAA3*	Forward Reverse	GAAGGAGATTGAATCATCATCAAGG GATTCCTTGACCCTCATGCTC
*IAA4*	Forward Reverse	GAAAGAGATTGAATCCACTGGA GTTTCCTTGACCTTCAGATTCAC
*IAA5*	Forward Reverse	GTAGATGGAGCTGCATTTTTAAG GGTACACATTCACTTTCCTTCAACG
*IAA6*	Forward Reverse	AGCATCGAAAGCTATAGGCTACG CTTCTTACCCTCCTTCGCCACT
*IAA11*	Forward Reverse	TTCCATGGCTGCAACTAGTGG CTTGTTTACGGCTTGACTTATCTCC
*IAA13*	Forward Reverse	CGGGTTAACCAGTCAGTTCAC TTAGCTTCAGAGGTTTTCATCACA
*IAA19*	Forward Reverse	TGGTGACAACTGCGAATACGTTAC CGTCTACTCCTCTAGGCTGCAG
*IAA20*	Forward Reverse	TCATGTTCAACGCATCCATTCTC CTTGAAATCTTCAGTCTTCTCACAG
*IAA29*	Forward Reverse	GGAATCCGAGTCTTCAATAGTTTACG ATGATACGGGCAATGATGGTG
*IAA30*	Forward Reverse	TCATGATTTGATCACAACTCTCGACTA GCTTTTAGAAAATCAGTAGTGATAAGC
*ACT2*	Forward Reverse	TTTTTCCCAGTGTTGTTGGTAGG GGTGCAAGTGCTGTGATTTCTTT
*ATHB8*	Forward Reverse	ATGGGAGGAGGAAGCAATAATAGTCACAAT TCATATAAAAGACCAGTTGAGGAACATGAAG
*ARF5-SfiI*	Forward Reverse	GGCCATTACGGCCATGATGGCTTCATTGTCTTG GGCCGAGGCGGCCGAAACAGAAGTCTTAAG
*ARF5CTD-SfiI*	Forward Reverse	GGCCATTACGGCCATGAATGTTGATTTTGATGATTG GGCCGAGGCGGCCGAAACAGAAGTCTTAAG

### Plant Transformation and Transgenic Plants Selection

To generate transgenic plants, about 5-week-old plants with several mature flowers on the main inflorescence were transformated via *Agrobacterium tumefaciens* (*GV3101*) using the floral dip method (Clough and Bent, 1998). To select transgenic plants, T1 seeds were sterilized and sown on 1/2 MS plates containing 50 μg ml^–1^ kanamycin. Phenotypes of transgenic plants were examined in the T1 generation, and confirmed in following generations. More than 10 transgenic lines with similar phenotypes were obtained. Homozygous T3 or T4 plants were used for the experiments.

### Auxin Treatment and Root Elongation Assay

For *PtrIAA14.1* expression analysis, leaves were collected from a 15-year-old field-grown poplar tree (Liu et al., 2013; Wang et al., 2014; Zhou et al., 2014), and leaf discs were prepared and treated with 10 μM IAA for 4 h in darkness on a shaker at 40 rpm. Samples were frozen in liquid N_2_, and kept in –80°C for RNA isolation.

For Aux/IAA gene expression analysis, 10-day-old wild-type and transgenic *Arabidopsis* seedlings grown on 1/2 MS plates were treated with 10 μM IAA for 4 h in darkness on a shaker at 40 rpm.

For root elongation and lateral root formation assays, 4-day-old wild-type and transgenic *Arabidopsis* seedlings grown on vertical plates were transferred onto new plates at the presence or absent of 0.1 μM IAA. Pictures were taken after growing vertically for additional 4 days, and root length was measured using ImageJ software.

### RNA Isolation and RT-PCR

Total RNA from poplar leaf discs was isolated as described previously (Geraldes et al., 2011; Liu et al., 2013; Wang et al., 2014). cDNA was synthesized using the Omniscript RT Kit (Qiagen) according to the manufacturer’s instructions.

Total RNA from *Arabidopsis* seedlings was isolated using Easy-Pure Plant RNA Kit (TransGen Biotech), and cDNA was synthesized using the EazyScript First-Strand DNA Synthesis Super Mix (TransGen Biotech) according to the manufacturer’s instructions.

All RT-PCR analyses were repeated at least three times and the representative images were presented. *Arabidopsis* gene *ACTIN2* (*ACT2*) and poplar gene *UBQ* were used as controls for RT-PCR.

### Yeast Two Hybridization

Split-ubiquitin system was used to detect the interaction between PtrIAA14.1 and ARF5. *ARF5CTD* was cloned into bait vector *pDHB1*, and *PtrIAA14.1* was cloned into prey vector *pPR3-N*. Bait and prey constructs were cotransformed into yeast strain NMY51. Cotransformation of empty bait and prey construct, and empty prey with bait construct were used as controls. The yeast transformants containing both prey and bait were able to grow on minimum synthetic dextrose dropout medium lacking both Trp and Leu (SD-trp-leu). A positive interaction between two proteins is indicated by the growth of yeast colony on the minimum synthetic dextrose medium lacking Leu, Trp, and His (SD-trp-leu-his), and a white color due to the activation of Ade synthesis.

### Protoplasts Isolation, Transfection, GUS Activity Assays, and Florescence Examination

Reporter and effector plasmids were prepared using the GoldHi EndoFree Plasmid Maxi Kit (CW Biotech) according to the manufacturer’s instructions. Protoplasts isolation, transfection, and GUS activity assays were performed by following the procedures described previously (Wang et al., 2005b; Tiwari et al., 2006). Florescence was examined under an Olympus BX-UCB/BX61 microscope.

### Vascular Patterning Observation

Cotyledons for vascular patterning observation were prepared as described by Krogan et al. (2012). Briefly, cotyledons from 10-day-old seedlings were fixed with ethanol: acetic acid (3:1, v/v) for 1 h, washed with 95% ethanol for 2 h, stored in 70% ethanol overnight, and then cleared in chloral hydrate: glycerol: water (8:3:1, w/v/v) overnight. Pictures were taken under an Olympus BX-UCB/BX61 microscope.

## Results

### PtrIAA14.1 is a Poplar Homolog of *Arabidopsis* IAA7

**FIGURE 1:**
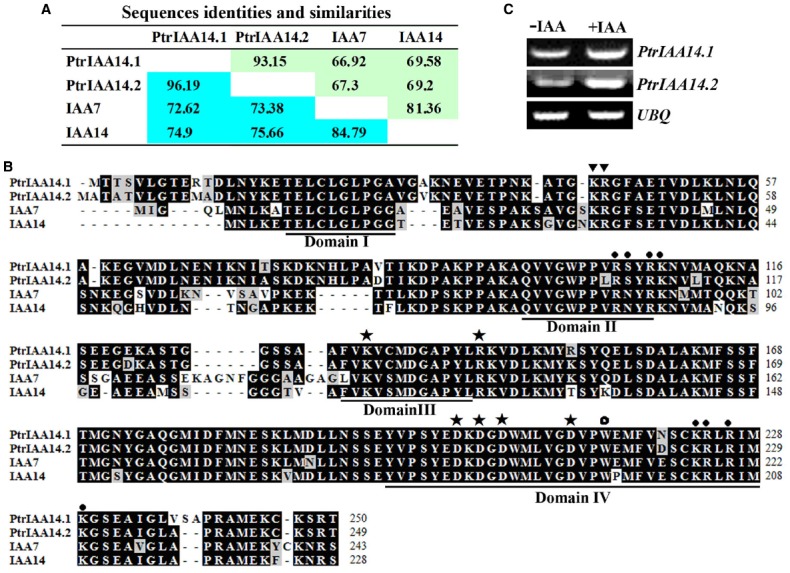
**PtrIAA14.1 is a homolog of *Arabidopsis* Aux/IAA protein IAA7. (A)** Amino acid sequence identities and similarities of poplar Aux/IAA proteins PtrIAA14.1 and PtrIAA14.2 and the *Arabidopsis* Aux/IAA proteins IAA7 and IAA14. Green stands for identities, and blue refers to similarities. **(B)** Amino acid sequence alignment of PtrIAA14.1, PtrIAA14.2, IAA7, and IAA14. The four conserved domains are underlined. Triangles indicate conserved KR residues between Domain I and Domain II that are crucial for protein degradation, closed circles indicate putative nucleus localization signals, asterisks indicate conserved residues crucial for protein–protein interaction, and open circle indicates conserved W residue in OsIAA23 that is crucial for protein-protein interaction. **(C)** Expression of *PtrIAA14.1* and *PtrIAA14.2* in poplar leaves in response to IAA treatment. RNA was isolated from IAA treated poplar leaf discs and RT-PCR was used to examine the expression of *PtrIAA14.1*. Expression of *UBQ* was used as a control.

PoptrIAA7.1 and PoptrIAA7.2 have been shown to be paralogs and are closely related to IAA7 and IAA14 (Kalluri et al., 2007). Because the poplar genome is still under fine annotation, we BLAST searched the updated poplar protein database (www.phytozome.net) using entire amino acid of IAA7 to confirm/identify its poplar homologs. We found that PoptrIAA7.1 and PoptrIAA7.2 are indeed homologs of IAA7 but they have been renamed PtrIAA14.1 (*Potri.008G161200*) and PtrIAA14.2 (*Potri.010G078300*), respectively. As shown in Figure 1A, the amino acid identity and similarity between IAA7 and PtrIAA14.1 are 66.92 and 72.62%, respectively, between IAA7 and PtrIAA14.2 are 67.3 and 73.38%, respectively, and between PtrIAA14.1 and PtrIAA14.2 are 93.15 and 96.19%, respectively.

PtrIAA14.1 and PtrIAA14.2 contain the four conserved domains found in most of the *Arabidopsis* Aux/IAA proteins including IAA7 and IAA14 (Figure 1B). In addition to the conserved LxLxL motif in Domain I, and the conserved QVVGWPPVRSYRK degron in Domain II (Tiwari et al., 2004; Dreher et al., 2006), amino acid sequence alignment showed that the predicted nucleus localization signal in *Arabidopsis* Aux/IAA proteins, the KR residues between Domain I and II that are crucial for 26 proteasome degradation of *Arabidopsis* Aux/IAA proteins (Dreher et al., 2006), the conserved amino acid residues in Domain III and IV that are require for homo and hetero interactions among Aux/IAA proteins and ARFs (Nanao et al., 2014), and the conserved W residue in OsIAAs and OsARFs that is crucial for protein–protein interaction (Ni et al., 2014) are all conserved in PtrIAA14.1 and PtrIAA14.2 (Figure 1B). Thus only PtrIAA14.1 was chosen for further functional characterization.

Expression of most *Arabidopsis* Aux/IAA genes including *IAA7* were auxin inducible. To test if *PtrIAA14.1* is also auxin inducible, leaf discs from poplar were treated with exogenously IAA, and RT-PCR was used to examine the expression levels of *PtrIAA14.1*. As shown in Figure 1C, the transcription level of both *PtrIAA14.1* and *PtrIAA14.2* was slight increased in response to auxin treatment.

### PtrIAA14.1 is a Transcription Repressor

Because PtrIAA14.1 is highly similar to IAA7 at amino acid sequence level, and it contains all the conserved motifs and amino acid signatures presented in IAA7 (Figure 1B), we examined if PtrIAA14.1 functions as transcriptional repressor as well with protoplast transfection assays.

**FIGURE 2:**
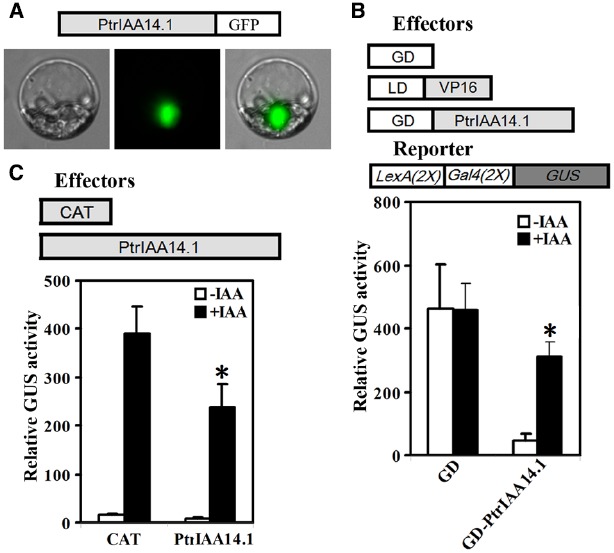
**PtrIAA14.1 is a transcriptional repressor. (A)** Nuclear localization of PtrIAA14.1. PtrIAA14.1-GFP (diagrammed on the top of the figure). Plasmids were transfected into protoplasts isolated from *Arabidopsis* leaves and incubated in darkness for 20–22 h before florescence was examined under a florescence microscope. Repression of *LexA-Gal4-GUS* reporter gene **(B)** and the integrated *DR5:GUS* reporter gene **(C)** by PtrIAA14.1. Effectors and reporters (diagrammed on the top of the figures) or effectors plasmids alone were transfected into protoplasts and incubated for 20–22 h before GUS activity was assayed. Data represent the mean ± SD of three replicates. *Significantly different from absence of IAA **(B)**, or CAT control **(C)** (*P* < 0.05).

We first tested if PtrIAA14.1 is a nuclear protein by expressing PtrIAA14.1-GFP fusion proteins in protoplasts. As shown in Figure 2A, florescence was observed predominately in nucleus. We then co-transfected N-terminal GAL4 DNA binding domain (GD) fused with PtrPtrIAA14.1 (GD-PtrIAA14.1) together with transcriptional activator LD-VP, and the *LexA-GAL4-GUS* reporter gene into protoplasts. Co-transfection of GD was used as a control. As shown in Figure 2B, in the absence of IAA, co-transfection of LD-VP and GD activated the reporter gene, while co-transfection of LD-VP and GD-PtrIAA14.1 resulted in repression of the reporter gene, suggesting that PtrIAA14.1 functions as a transcriptional repressor. Application of auxin partially released the repression on the expression of the reporter gene by PtrIAA14.1 (Figure 2B), indicating that PtrIAA14.1, similar to IAA7, is unstable in the presence of auxin. We also examined if PrIAA14.1 represses auxin response gene expression by expressing PtrIAA14.1 in protoplasts containing an integrated auxin response gene reporter *DR5:GUS*. As shown in Figure 2C, PtrIAA14.1 repressed *DR5:GUS* expression.

### Expression of *PtrIAA14.1* Resulted in Morphological Changes in *Arabidopsis*

To further explore the function of PtrIAA14.1 in auxin signaling and plant growth and development, we generated transgenic plant expressing *PtrIAA14.1* under the control of the *35S* promoter. Because PtrIAA14.1 contains a wild-type Domain II, we were not expecting to see any morphological alternations in the transgenic plants if PtrIAA14.1 acts similarly as *Arabidopsis* Aux/IAA proteins. However, more than 10 lines of transgenic plants with similar phenotypic changes were obtained. Because fertility in strongest transgenic lines was abolished, two moderate transgenic lines were selected for further phenotypic analysis.

**FIGURE 3:**
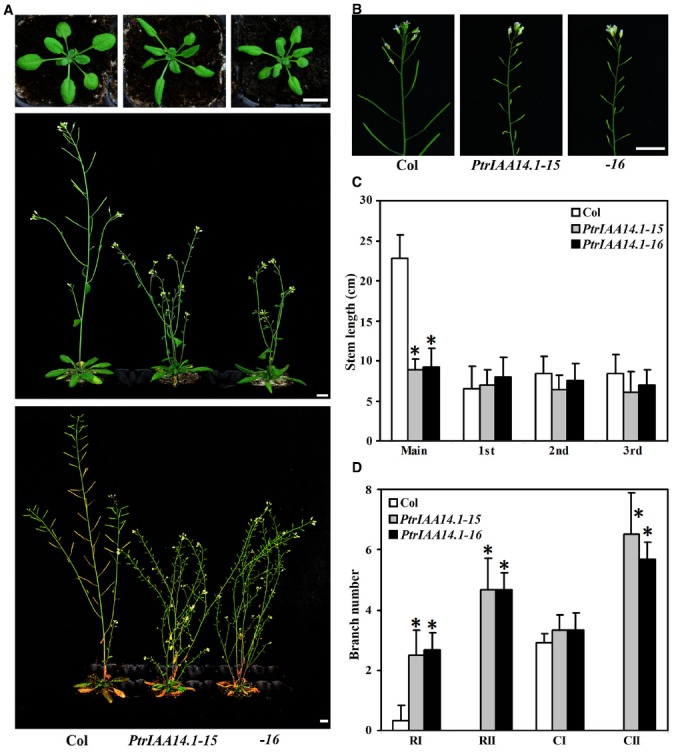
**Morphological comparison of wild-type plants and transgenic *Arabidopsis* plants expressing wild-type *PtrIAA14.1*. (A)** Three-week-old (top panel), 5-week-old (middle panel), and 7-week-old (bottom panel) wild-type and transgenic plants. **(B)** Inflorescence stems from 5-week-old wild-type and transgenic plants. **(C)** Stem length of wild-type and transgenic plants. Length of main and branch stems from 5-week-old plants was measured. **(D)** Branch number of wild-type and transgenic plants. Branches on 7-week-old were counted. The names used for different branches were as described by Aguilar-Martínez et al. (2007). Scale bars: **(A,B)**, 1 cm. Data in **(C,D)** represent the mean ± SD of 5–11 plants. *Significantly different from wild-type (*P* < 0.01).

As shown in Figure 3, adult transgenic plants expressing *PtrIAA14.1* showed several auxin-related phenotypes, including down-curling leaves, semi-draft with increased number of branches (Figure 3A), and greatly reduced fertility (Figure 3B). Quantitative analysis showed that the length of main inflorescence stems in transgenic plants was greatly reduced, whereas length of branch stems on the main inflorescences was largely unaffected (Figure 3C). Our analysis also showed that when compared with that in wild-type plants, the number of primary rosette-leaf branch (RI) was increased dramatically, while the number of primary cauline-leaf branch (CI) remained unchanged in the transgenic plants (Figure 3D). On the other hand, under our growth conditions, wild-type plants did not, but transgenic plants produced secondary rosette-leaf branch (RII) and secondary branch cauline-leaf (CII; Figure 3D).

### Root Elongation in *Arabidopsis* Seedlings Expressing *PtrIAA14.1* is Less Sensitive to Auxin Treatment

**FIGURE 4:**
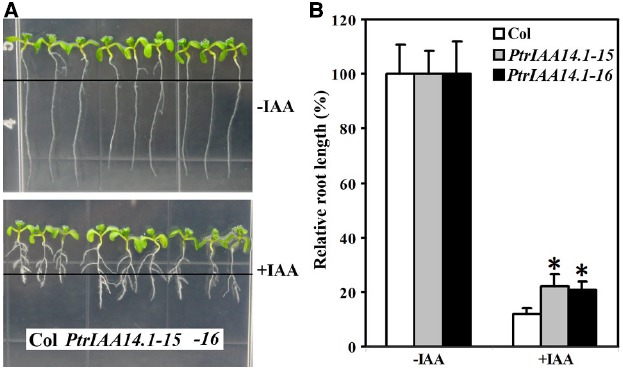
**Effects of auxin on root elongation and lateral root formation in wild-type and transgenic plants. (A)** Eight-day-old seedlings on vertical plates at the presence (bottom panel) and absent (top panel) of 0.1 μM IAA. Four-day-old seedlings grown on vertical plates were transferred into new plates with or without auxin added, and grown vertically for additional 4 days. Lines indicate the root tip positions immediately after transfer. **(B)** Inhibition of root elongation by auxin. Length of new elongated roots was measured and relative root length was calculated. Data represent the mean ± SD of at least 10 seedlings. *Significantly different from wild-type (*P* < 0.0001).

Though pleiotropic phenotypes were observed in adult transgenic plants expressing *PtrIAA14.1* (Figure 3), transgenic plant seedlings were largely indistinguishable from wild-type (Figure 4A). To examine if auxin responsiveness in transgenic plants is altered, we analyzed the effects of auxin on root elongation and lateral root formation in transgenic plant seedlings.

Four-day-old seedlings of wild-type and transgenic plants grown on vertical 1/2 MS plates were transferred to new 1/2 MS plates with or without 0.1 μM IAA and grown vertically for additional 4 days. The root length was measured and relative root growth was calculated. As shown in Figure 4, *Arabidopsis* seedlings expressing PtrIAA14.1 was less sensitive to auxin treatments. The root length of wild-type seedlings with auxin treatments averaged about 10% of the root length (i.e., reduction by 90%) as compared to seedlings grown in normal conditions. In comparison, PtrIAA14.1 seedlings with auxin treatments grew to about 20% of the normal root length (i.e., reduction by 80%; Figure 4B). Lateral root formation in transgenic plant seedlings in response to auxin, however, remains unchanged when compared with that in wild type (Figure 4A).

### Expression of the *Arabidopsis Aux/IAA* genes in Transgenic Plants is Largely Unaffected

**FIGURE 5:**
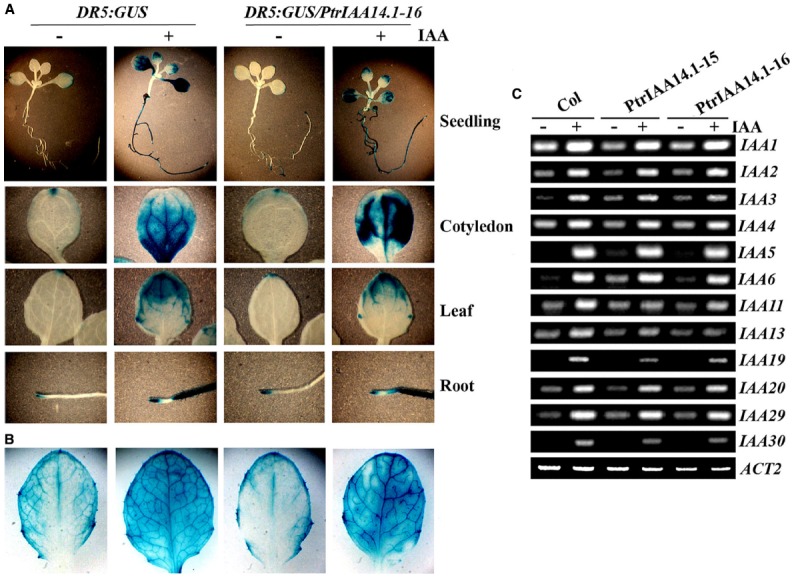
**Expression of auxin response genes in transgenic plants expressing *PtrIAA14.1*.** Expression of *DR5:GUS* reporter gene in wild-type and transgenic seedlings **(A)** and mature rosette leaves from 3-week-old plants **(B)**. X-Gluc (5-bromo-4-chloro-3indolyl-*β*-d-glucuronide) was used for histochemical staining of GUS activity. **(C)** Expression of *Aux/IAA* genes in wild-type and transgenic plants. Ten-day-old seedlings were treated with 10 μM IAA for 4 h, and then RNA was isolated. RT-PCR was used to examine the expression of *Aux/IAA* genes. Expression of *ACT2* was used as a control.

PtrIAA14.1 repressed auxin response reporter gene expression in protoplasts (Figure 2C). Adult transgenic plants expressing *PtrIAA14.1* have pleiotropic phenotypes (Figure 3), and was less sensitive to auxin treatments as measured by the root growth inhibition assays (Figure 4). We wanted to further examine if expression of auxin responsive genes in the transgenic plants was affected. We first crossed *DR5:GUS* reporter line to the *PtrIAA14.1* transgenic line, and examined reporter gene expression by detecting GUS activity in the seedlings. As shown in Figure 5A, in both wild-type and transgenic plant background, GUS activity was mainly observed in root and cotyledon tips in the absent of auxin treatment, and GUS activity was detected in nearly all parts of the seedlings when treated with 10 μM IAA, however, no dramatically difference was observed between wild-type and transgenic plants. On the other hand, slightly increased GUS activity was observed near the edges of full expanded rosette leaves from 3-week-old transgenic plants, and in the veins of the leaves upon auxin treatment (Figure 5B).

By using RT-PCR, we also examined the expression of some endogenous Aux/IAA genes in the transgenic plants, including *IAA1* to *6*, *IAA11*, *IAA13*, *IAA19*, *IAA20*, *IAA29*, and *IAA30*. Expression of all the *Arabidopsis* Aux/IAA genes tested was elevated in response to auxin treatment, and in general, their expression in transgenic plants remains largely unchanged (Figure 5C). Yet some slight changes in the expression of some *Aux/IAA* genes were observed in the transgenic plants. For example, the expression of *IAA6* was slightly increased in the transgenic plants in the absent of auxin treatment, and auxin responsiveness of *IAA11* was slightly decreased in the transgenic plants (Figure 5C).

### PtrIAA14.1 Interacts With ARF5 in Yeast and in Plant Cells

Interplay of ARFs and Aux/IAA proteins controls auxin signaling in *Arabidopsis*. There are only five ARFs including ARF5, ARF6, ARF7, ARF8, and ARF19, that are transcription activators. Because that PtrIAA14.1 repressed auxin response reporter gene expression in protoplasts (Figure 2C), and transgenic plants expressing *PtrIAA14.1* have auxin-related phenotypes (Figure 3), we further examined if PtrIAA14.1 regulates auxin signaling and plant growth and development via interacting with ARF activators by using yeast two hybridization and protoplast transfection assays.

**FIGURE 6:**
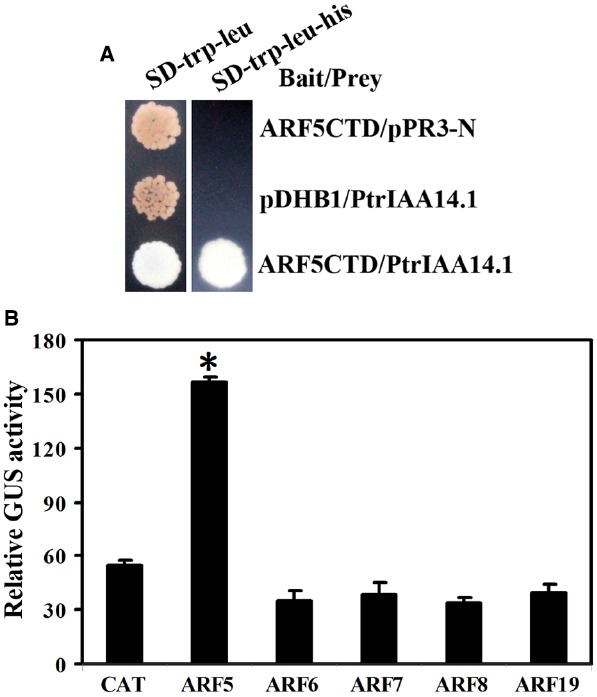
**Interaction between PtrIAA14.1 and ARF5 in yeast and plant cells. (A)** PtrIAA14.1 interacts with ARF5 in yeast. Bait and prey plasmids were cotransformed into NMY51 yeast cells and grown in SD-trp-Leu and SD-trp-leu-his plates for 2–4 days before picture were taken. **(B)** PtrIAA14.1 interacts with ARF5 in protoplasts. PtrIAA14.1CTD and ARF5 plasmids were co-transfected into protoplasts and incubated for 20–22 h before GUS activity was assayed. Data represent the mean ± SD of three replicates. *Significantly different from CAT control (*P* < 0.0001).

Empty bait vector *pDHB1* or bait vector containing ARF activator CTD coding sequences and prey vector containing full-length PtrIAA14.1 coding sequence or empty prey vector *pPR3-N* were cotransformed into yeast strain *NMY51*, and grown in SD-trp-leu and SD-trp-leu-his plates. The results showed that only yeast transformed with bait vector containing ARF5CTD and prey vector containing full-length PtrIAA14.1 grew on SD-trp-leu-his plates (Figure 6A), indicating that PtrIAA14.1 interacts with ARF5 in yeast.

To confirm the interaction of PtrIAA14.1 and ARF5 in planta, plasmids of GD fused PtrIAA14.1CTD (GD-PtrIAA14.1CTD) and ARF activators were cotransfected with *Gal4:GUS* reporter into protoplasts, and GUS activity was measured after incubation. As shown in Figure 6B, cotransfection of GD-PtrIAA14.1CTD with ARF5, but not ARF6, ARF7, ARF8, and ARF19 activated Gal4-GUS reporter, suggesting that PtrIAA14.1 only interacts with ARF5.

### Vascular Patterning is Altered in the PtrIAA14.1 Transgenic Plants

**FIGURE 7:**
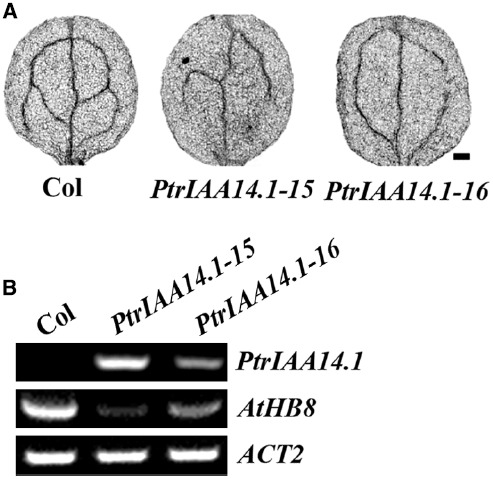
**Expression of *PtrIAA14.1* in *Arabidopsis* affected vascular development. (A)** Vascular patterning in wild-type and transgenic plant cotyledons. Cotyledons were treated with ethanol: acetic acid (3:1, v/v) solution for 1 h, dehydrated in 95% ethanol for 2 h and in 70% ethanol for overnight, and then cleared in chloral hydrate: glycerol: water (8:3:1, w/v/v) overnight before the pictures were taken. Scale bar: 20 μm. **(B)** Expression of *AtHB8* in transgenic plants. RNA was isolated from 10-day-old seedlings and RT-PCR was used to examine the expression of *AtHB8*. Expression of *ACT2* was used as a control.

It has been reported that ARF5 regulates vascular development in *Arabidopsis* (Hardtke and Berleth, 1998; Donner et al., 2009). Our results in yeast and protoplast cells suggest that PtrIAA14.1 interacts with ARF5. We hypothesized that vascular patterning in transgenic plants expressing PtrIAA14.1 may be affected. As shown in Figure 7A, changes of vascular patterning were also observed in full expanded rosette leaves (Figure 5B).

Previously studies showed that *AtHB8 (Arabidopsis thaliana homeobox gene 8*) is an important regulator of vascular development (Baima et al., 2001), and that ARF5 regulates vascular patterning through activating *AtHB8* (Donner et al., 2009). Thus we further examined the expression of *AtHB8* in *PtrIAA14.1* transgenic plants. We found that, unlike the *Aux/IAA* genes tested, expression of *AtHB8* was dramatically decreased in the transgenic plants (Figure 7B), suggesting that PtrIAA14.1 regulates vascular development in *Arabidopsis* by repressing the expression of *AtHB8*.

## Discussion

*Arabidopsis* Aux/IAA proteins are transcription repressors that negatively regulate auxin response gene expression by interacting with ARFs (Guilfoyle, 1999; Hagen and Guilfoyle, 2002). Characterization of dominant gain-of-function mutants revealed that *Aux/IAA* genes regulate many aspects of plant growth and development (Liscum and Reed, 2002). Yet the functions of Aux/IAA proteins in other plants remain largely unknown. Our results in this report showed that poplar Aux/IAA protein PtrIAA14.1 regulates auxin signaling and plant growth and development in *Arabidopsis* in a way similar but may also be different from that of *Arabidopsis* Aux/IAA proteins.

### PtrIAA14.1 is a Classic Aux/IAA Protein

Most Aux/IAA proteins including IAA7 in *Arabidopsis* contain four conserved domains, namely Domain I, Domain II, Domain III, and Domain IV. Domain I contains a LxLxL repression motif, Domain II contains a degron, Domain III and IV contain conserved amino acid residues that are required for protein–protein interactions (Ulmasov et al., 1997, 1999; Ramos et al., 2001; Tiwari et al., 2001, 2003, 2004; Dreher et al., 2006; Nanao et al., 2014).

In poplar, there are 35 genes encoding Aux/IAA proteins. PtrIAA14.1 is the most closely related one to *Arabidopsis* IAA7 (Kalluri et al., 2007). Bioinformatics analysis showed that PtrIAA14.1 shared high sequence identity and similarity to IAA7 on protein sequence level (Figure 1A), and that it contains all of the Aux/IAA features described above (Figure 1B). In addition, PtrIAA14.1 also contains the conserved KR residues that have been shown to be crucial for 26 proteasome degradation of Aux/IAA proteins (Dreher et al., 2006), and the conserved W residue in OsIAAs and OsARFs that has been shown to be crucial for protein–protein interactions (Ni et al., 2014; Figure 1B). Thus we expect PtrIAA14.1 may have similar functions to most *Arabidopsis* Aux/IAA proteins do. Indeed, protoplast transfection assays showed that PtrIAA14.1 was localized in nucleus. It was unstable in the presence of auxin, and it functioned as a transcription repressor. Moreover, it repressed auxin response reporter gene expression (Figure 2). All these results suggest that PtrIAA14.1 is a classic Aux/IAA protein.

### Similarity and Specificity of PtrIAA14.1 and *Arabidopsis* Aux/IAA Proteins in Regulating Plant Growth and Development

The functions of *Arabidopsis* Aux/IAA proteins in the regulating of plant growth and development are revealed by characterization of dominant gain-of-function mutants, and all the mutations were occurred within Domain II of corresponding Aux/IAA proteins (Tian and Reed, 1999; Nagpal et al., 2000; Rogg et al., 2001; Fukaki et al., 2002; Tian et al., 2002; Knox et al., 2003; Uehara et al., 2008; Ploense et al., 2009; Rinaldi et al., 2012), indicating that stability of Aux/IAA proteins are crucial for their proper functions in auxin regulated plant growth and development. Consistent with this, transgenic *Arabidopsis* plants overexpressing wild-type Aux/IAA1 are morphological indistinguishable to wild type (Park et al., 2002). Transgenic *Arabidopsis* plants expressing a wild-type grape Aux/IAA are also morphological similar to wild-type plants (Fujita et al., 2012; Kohno et al., 2012). Whereas transgenic *Arabidopsis* plant overexpressing Domain II mutated IAA1, IAA 14, Domain II lacking Aux/IAA, or Aux/IAA with dominant mutation-type Domain II resulted in auxin-related phenotypes (Park et al., 2002; Fukaki et al., 2005; Sato and Yamamoto, 2008).

Because PtrIAA14.1 is a classic Aux/IAA protein with all the features presented in most *Arabidopsis* Aux/IAA proteins, including a degron in Domain II, and conserved KR residues between Domain I and Domain II (Figure 1B), and PtrIAA14.1 was unstable in protoplasts in the presence of auxin (Figure 2B), we did not expected that expression of *PtrIAA14.1* in *Arabidopsi*s would result in any morphological changes. To our surprise, transgenic *Arabidopsis* plants expressing *PtrIAA14.1* showed several auxin-related phenotypes (Figure 5), and were less sensitive to auxin in term of root elongation (Figure 4). Yet the phenotypes are largely different from that in the dominant gain-of-function mutant *iaa7* (Nagpal et al., 2000). On the other hand, the expression of most of the *Arabidopsis Aux/IAA* genes tested in the transgenic plants remained largely unchanged. These results indicate that unlike most *Arabidopsis* Aux/IAA proteins, PtrIAA14.1 may use different mechanisms in regulating plant growth and development.

### PtrIAA14.1 Regulates Vascular Patterning in *Arabidopsis* Through Interacting with ARF5

Aux/IAA proteins regulate auxin response gene expression via interacting with ARFs (Guilfoyle and Hagen, 2007). PtrIAA14.1 has all the conserved amino acids required for protein–protein interactions in Domain III and IV (Nanao et al., 2014). It also has the W residue conserved in OsIAAs and OsARFs that is required for protein–protein interactions (Ni et al., 2014). Thus it is reasonable to expect that PtrIAA14.1 interacts with ARFs. Indeed, interaction between PtrIAA14.1 and ARF5 was observed in both yeast and plant cells (Figure 6).

Previously studies have shown that ARF5 regulates vascular development by activating *AtHB8* (Donner et al., 2009). Our results also showed that vascular patterning was changed in the transgenic plants expressing *PtrIAA14.1*, and that the expression of *AtHB8* in transgenic plants was also downregulated, suggesting that PtrIAA14.1 regulates vascular development when expressed in *Arabidopsis* by repressing the expression of *AtHB8* via interacting with ARF5. Interaction of PtrIAA14.1 with ARF5, but not other ARF activators (Figure 6B) may also responsible for the auxin-related phenotypes observed in the transgenic plants expressing *PtrIAA14.1*.

Because there is only one amino acid residue difference found in Domain III and Domain IV, respectively, between PtrIAA14.1 and IAA7, and the total number of amino acid residue difference in the C-terminal of PtrIAA14.1 and IAA7 was less than 10 (Figure 1B), it will be interesting to examine why PtrIAA14.1 interacts specifically with ARF5.

### Conflict of Interest Statement

The authors declare that the research was conducted in the absence of any commercial or financial relationships that could be construed as a potential conflict of interest.
